# LONG-TERM OUTCOMES OF USING VARIOUS GRAIN ALLOGRAFT SIZES IN PAPROSKY TYPE 3

**DOI:** 10.1590/1413-785220243202e273746

**Published:** 2024-06-24

**Authors:** Patcharavit Ploynumpon, Rutthakorn Sritumma, Thakrit Chompoosang

**Affiliations:** 1 Rajavithi Hospital, Faculty of Medicine, Department of Orthopedics, Ratchathewi District, Bangkok, Thailand.

**Keywords:** Arthroplasty, Replacement, Hip, Allografts, Surgical Procedures, Operative, Artroplastia de Quadril, Aloenxertos, Procedimentos Cirúrgicos Operatórios

## Abstract

**Introduction:**

Severe acetabular bone defects can pose challenges in revision total hip replacement. The use of structural allografts and various sizes of grain allografts has been proposed as an alternative surgical technique for treating Paprosky type 3 acetabular defects. This study aimed to evaluate the long-term outcomes and potential complications associated with this approach.

**Methods:**

A retrospective review was performed on 102 hip reconstructions in patients with major acetabular bone loss, including 81 cases of type 3A and 21 cases of type 3B according to Paprosky's classification. Surgical procedures involved the use of structural allografts and various sizes of grain allografts in both reinforcement ring group and cementless cups group.

**Results:**

At a mean follow-up of 82.75 months, 76% of hips had no complications, while The others experienced pain changes in the cup position, post-operative dislocations, and infections. The mean pre-operative Modified Harris Hip Score improved in both groups at the last follow-up.

**Conclusion:**

The use of structural allografts and various sizes of grain allografts for treating type 3 acetabular defects in revision total hip replacement showed promising long-term outcomes and a low rate of complications. ***Level of Evidence IV; Retrospective Case Series.***

## RESUMO

**Introdução:**

Defeitos ósseos acetabulares graves podem representar desafios na revisão da artroplastia total do quadril. O uso de aloenxertos estruturais e aloenxertos de grãos de vários tamanhos foram propostos como uma técnica cirúrgica alternativa para o tratamento de defeitos acetabulares Paprosky tipo 3. O objetivo deste estudo foi avaliar os resultados de longo prazo e as possíveis complicações associadas a essa abordagem.

**Métodos:**

Foi realizada uma revisão retrospectiva de 102 reconstruções de quadril em pacientes com grande perda óssea acetabular, incluindo 81 casos do tipo 3A e 21 casos do tipo 3B de acordo com a classificação de Paprosky. Os procedimentos cirúrgicos envolveram o uso de aloenxertos estruturais e aloenxertos de grãos de vários tamanhos, tanto no grupo do anel de reforço quanto no grupo das próteses sem cimento.

**Resultados:**

Em um acompanhamento médio de 82,75 meses, 76% dos quadris não apresentaram complicações, enquanto os demais apresentaram dor, alterações na posição da prótese, luxações pós-operatórias e infecções. A pontuação média pré-operatória do escore de quadril modificado de Harris melhorou em ambos os grupos no último acompanhamento.

**Conclusão:**

O uso de aloenxertos estruturais e aloenxertos de grãos de vários tamanhos para o tratamento de defeitos acetabulares do tipo 3 na substituição total do quadril de revisão mostrou resultados promissores em longo prazo e uma baixa taxa de complicações. ***Nível de Evidência IV; Série de Casos Retrospectivos.***

## INTRODUCTION

Severe acetabular bone defects can present a challenging problem in revision total hip replacement. There are many treatment options available for managing acetabular bone defects, including the use of structural allografts from the distal femur, proximal tibia, and femoral head, combined with cemented or cementless cups or acetabular reinforcement rings. While these methods provide relatively good short-term results, the failure rate for mid and long-term outcomes can range between 4% and 47%.^1^

Another method proposed by Lebeau et al. involved the use of a dual-mobility acetabular cup cemented in a metal reinforcement (reconstruction acetabular ring) with bone graft filling the defect in revision total hip arthroplasty with severe acetabular bone defects and a high risk of dislocation. This approach provided good mid-term outcomes, with a survival rate of 91.9% for an 8-year follow-up period.^2^

In our study, we aimed to evaluate an alternative bone graft technique that involves the use of structural allografts and various sizes of grain allografts for treating type 3 acetabular defects according to Paprosky's classification, with a focus on assessing the long-term outcomes and potential complications associated with this approach.

## METHODS

A retrospectively reviews was performed in 102 hip reconstructions (101 patients) associated with major acetabular bone loss were conducted after received approval from the Ethics board Committee and Informed consent form(ICF) were signed by all participants. There were 81 hips in type 3A and 21 hips in type 3B according to Paprosky's classification. Pelvic discontinuity was 28 cases. The series included 52 right and 51 left with mean age of 57.3 years (34-83). All cases performed acetabular reconstruction from 2008 to 2019. There were 62 for aseptic loosening, 7 for protusio acetabuli post-hemiarthroplasty, 21 for second-stage revision after infected total hip arthroplasty, 5 for primary osteoarthritis, and 7 total hip arthroplasty instability.

**Figure 1:**
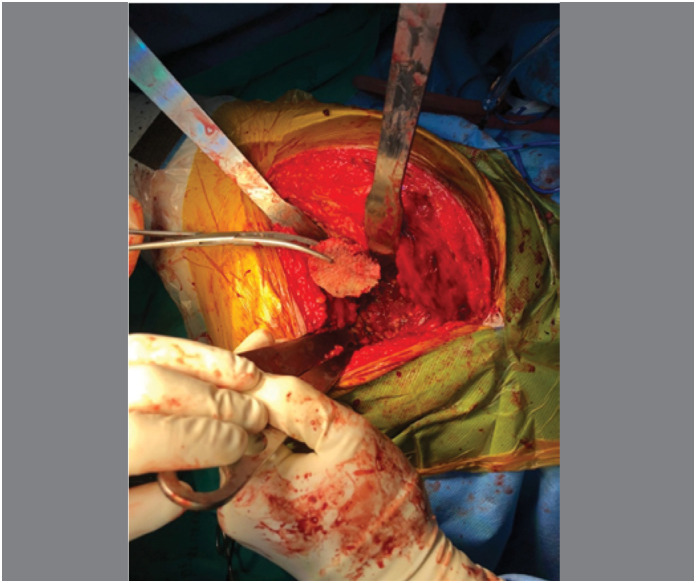
Structural allografts was placed into cavity at superoposterior part or the medial wall defect.

**Figure 2:**
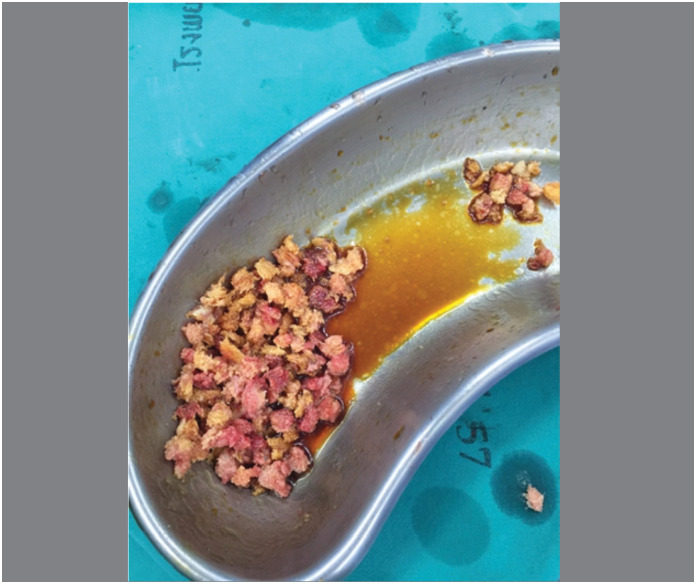
Using Various or multiple Grain sizes bone graft.

**Figure 3:**
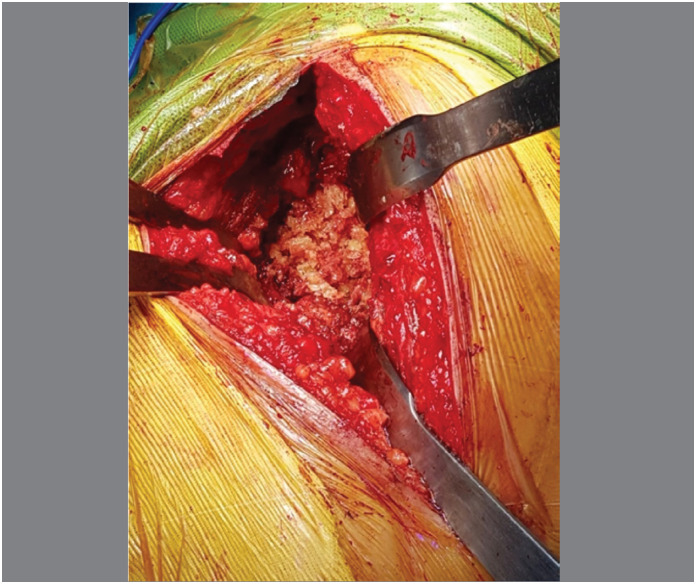
Grain bone graft were impacted into medial defect.

**Figure 4:**
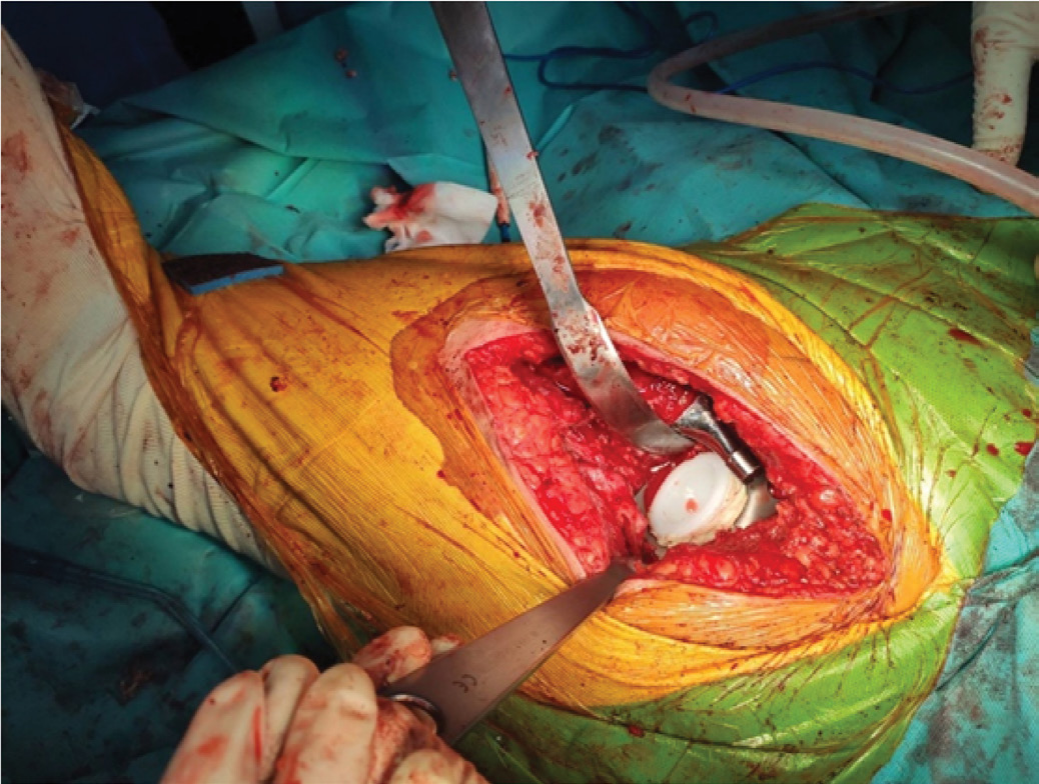
After gone graft was impacted, reconstruction cage + cemented cup was placed.

Surgical procedures: The acetabular reconstructions were performed by structural allografts placed into cavity at super posterior part or the medial wall defect (Figure 1), then two sizes of grain bone graft by bone mill machine (Tracer design) were filled in the space(Figure 2 and 3). After all allografts were placed, 42 cases were performed with reinforcement ring and cemented cup, 60 cases with a cementless cup (Figure 4).

### Statistical analysis

The study analyzed data using the SPSS Statistics program(IBM Corporation, Armonk, New York, USA), Version 22and the follow-up period for the participants was defined as the time between the acetabular component implantation and reoperation related to the component, death, or the end of the follow-up period. The Descriptive data was presented as median, minimum, maximum, and percentage values. The survival analysis was assessed on the Kaplan-Meier method.

## RESULTS

**Figure 5:**
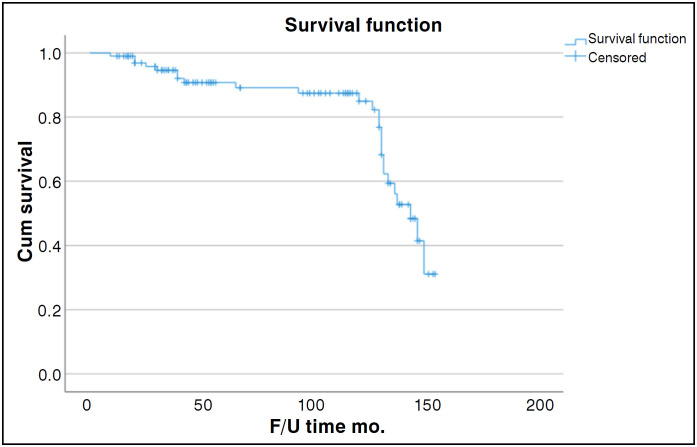
Survival of the acetabular implant.

One-hundred two hip reconstructions were performed (One-hundred one patients). Overall mean follow-up of 82.75 months (9 to 154), seventy-eight hips (76%) had no complications(Figure 5).

In the cementless cup group, one patient (1%) needed revision with a basic cementless cup after two years due to discomfort from a conspicuous implant. Two cases (2.1%) had the cup position change, but there were no symptoms and no revisions required. One hip (1.6% of all hips) had post-operative dislocations; this was addressed with open reduction and trochanter fixation. Two hips (2%) had deep infection after surgery; these were managed with debridement, and two case were lost follow up. There were two cases of deaths from congestive heart failure and ischemic heart disease. At the most recent follow-up, the mean pre-operative Modified Harris Hip Score increased from 28.3 points (range 16–44) to 89.1 points (range 73–95).

In the group of patients who received a reinforcement ring and cemented cup, only one hip (1%) had a change in the cup's position. However, it did not result in any symptoms or need a revision procedure. Two hips (3.2%) had post-operative dislocations, which were addressed by cup revision. Five hips (5%) had a deep infection after surgery, which was managed with a two-stage revision arthroplasty. Four follow-up cases were lost. Two cases were death from heart failure and pneumonia. The average pre-operative Modified Harris Hip Score increased from 22.7 points (13-39) to 82.1 points (60-89) at the final follow-up.(Table 1)

**Table 1:** Summary of the result of the study.

Result	Cementless cup (hip)	Reinforcement ring with cemented cup (hip)
Revision Rate	1	1
Cup position change	2	1
Post operative dislocation	1	2
Deep infection	2	5
Death	2	2
Follow up loss	2	4
Pre-operative Harris hip score (Mean +/-SD)	28.3 ± 8.2	22.7 ± 7.1
Post-operative Harris Hip Score (Mean SD)	89.1 ± 5.2	82.1 ± 8.4

## DISCUSSION

Acetabular reconstruction is a surgical procedure used to rebuild the acetabulum, or the socket of the hip joint, in cases where there has been significant bone loss which is typically performed in patients who have experienced aseptic loosening, protusio acetabuli or revision after infected total hip arthroplasty.

There are a number of techniques available for restoring acetabular bone loss, including the use of structural allografts, cementless hemispherical cups, oblong cups, extra-large cups, modular porous augments, impaction bone grafting (IBG), reinforcement rings. Which depended on degree of bone loss, patient status, surgeon preference. However, Many study shown that biological reconstruction using impaction bone grafting has the added advantage of improving and potentially restoring bone stock for future revisions and has favorable longevity (85%-90% survival rate of implants).^3^ But, the downside of this technique were technical demanded, risk of graft resorption infection and time consuming.^4^

When compare to the technique as in the study by Perlbach et al., the use of extensive bone impaction grafting in combination with an uncemented component in acetabular revisions resulted in good implant survival rates of 96.3% (95% CI 94.1 to 98.5) after ten years and 92.8% (95% CI 89.2 to 96.6) after 15 years in a sample of 370 patients.^5^ The implementation of tantalum augmentation as a viable alternative to allograft bone in the management of acetabular defects provides several advantages. Its high coefficient of friction and porous structure, like trabecular bone, impart stability and foster bone and fibrous ingrowth. The ability to tailor the various shapes and sizes of tantalum augments to specific defects also contributes to a reduction in operative time.^6^ The mid-term outcomes of the utilization of tantalum augmentation in conjunction with cement cups and cages, as reported by Mahmoud et al, demonstrate favorable results with survivorship rates of 95.8% at a median follow-up of 5 years and 97.2% at a mean follow-up of 60.1 months.^7^ However, the study of Qiang Xiao et el found that 4.9% of patients had a high hip center with measurements of 35.9 mm and 44.2 mm. The success of the results was attributed to restoring the hip center to normal biomechanics, as a high hip center can impact the function of the abductor muscles, and a longer neck length can mitigate this impact.^8^ Our case series assessed the use of a combination of structural allograft and different size impaction grafted bone allografts in the treatment of Paprosky type 3 bone deficiency, a severe bone defect. Complication rates did not differ significantly between the cementless and cemented cup groups in our study, which utilized grafts of varying sizes. This finding is noteworthy because it suggests that our grafting technique can be used in a range of scenarios, contingent on the patient's unique anatomy and the surgeon's needs, and that it can produce favourable outcomes with respect to complications, survival rate and Herris hip score.

Other than Being the first study to investigate the use combination of structural allograft and various grain allograft as a technique for improving outcomes, providing an alternative to traditional allograft use. This study has several strengths, including a comprehensive, long-term follow-up period based on registered data and the consistent application of the surgical technique by a small team of highly experienced surgeons.

However, This study has a number of limitations due to its retrospective design, moderate lost follow up and mortality rate, which hindered the ability to complete follow-up for all participants and some of the data were missing which can affect statistical analysis.

## CONCLUSION

The goal of using allografts in the treatment of acetabular bone defects is to restore bone stock for stability in primary or revision hip replacement surgeries. The combination of structural allografts and grain allografts of various sizes can be effective in achieving this goal. The larger particles provide improved mechanical stability and better vascularization and cement penetration, while the smaller particles fill in the spaces between the larger particles and facilitate ongoing biological healing.

Based on our long-term results, it appears that acetabular reconstruction using a combination of structural allografts and various sizes of grain allografts is effective in the treatment of Paprosky type 3 bone deficiency.
